# High-Sensitivity Cardiac Troponin I (hs-cTnI) Levels Increase After Radiofrequency Ablation and Are Associated with Procedural Success in Left Ventricular Summit-Derived Premature Ventricular Complex Ablation

**DOI:** 10.3390/jcm15145652

**Published:** 2026-07-18

**Authors:** Fadime Koca, Atilla Bulut, Yurdaer Donmez, Hilmi Erdem Sumbul, Mevlut Koc

**Affiliations:** 1 Department of Cardiology, Cukurova State Hospital, Adana 01170, Turkey; drfadimekoca@gmail.com; 2 Department of Cardiology, University of Health Sciences, Adana Health Practice and Research Center, Dr. Mithat Özsan Bulvarı Kışla Dist., 4522 Street No: 1, Yüreğir, Adana 01230, Turkey; atilabulut@gmail.com (A.B.); yurdaerd@gmail.com (Y.D.); 3 Department of Internal Medicine, University of Health Sciences—Adana Health Practice and Research Center, Adana 01230, Turkey

**Keywords:** premature ventricular complex, left ventricular summit, hs-cTnI, radiofrequency ablation

## Abstract

**Introduction**: There are no data in the literature regarding changes in cardiac troponin levels following radiofrequency ablation (RFA) in patients undergoing ablation for left ventricular summit-derived premature ventricular complexes (LVS-PVCs). In this study, we aimed to investigate changes in high-sensitivity cardiac troponin I (hs-cTnI) levels after RFA in patients with LVS-PVCs and to evaluate the clinical utility of hs-cTnI in this setting. **Method:** In this retrospective cohort study, 109 patients with LVS-PVCs who underwent RFA between 2017 and 2025 were included. In addition to routine evaluations, hs-cTnI levels were measured 24 h after the procedure in all patients. Long-term procedural success was assessed at 6 months using 24 h Holter electrocardiography. Patients were divided into two groups according to procedural outcome (successful vs. unsuccessful RFA). **Results:** In patients with LVS-PVCs, the long-term procedural success rate of RFA was 72.5% (*n* = 79). Compared with patients with unsuccessful long-term outcomes, those with successful RFA had significantly higher hs-cTnI levels, longer QRS-duration, a higher prevalence of RBBB morphology, more frequent ablation at the left coronary cusp (supravalvular/subvalvular), coronary sinus sites (GCV/AIV), and multiple ablation sites, as well as higher maximum RFA power. In contrast, the maximum deflection index and the presence of a pattern break in lead V2 were significantly lower in patients with long-term procedural success. In logistic regression analysis, hs-cTnI level, maximum RFA power, and ablation within the GCV/AIV were independently associated with RFA success (OR = 1.133, 95% CI: 1.043–1.231, *p* < 0.001; OR = 1.446, 95% CI: 1.176–1.773, *p* < 0.001; and OR = 3.281, 95% CI: 1.325–21.219, *p* = 0.002, respectively). ROC curve analysis demonstrated that hs-cTnI and maximum RFA power thresholds of 700 ng/L and 40 W, respectively, predicted RFA success with acceptable sensitivity and specificity. **Conclusions:** In patients with LVS-PVCs, hs-cTnI levels increase after RFA. Higher hs-cTnI levels are independently associated with procedural success. Measurement of hs-cTnI at 24 h after RFA may serve as an objective and noninvasive marker of procedural success in patients with LVS-PVCs.

## 1. Introduction

Premature ventricular complexes (PVCs) are a common cause of ventricular arrhythmias, with a reported prevalence of 1–4% in the general population, accounting for approximately 10–12% of all arrhythmia-related clinical presentations [1,2,3,4,5]. Although PVCs are generally considered to have a benign prognosis, a subset of patients may develop cardiomyopathy and life-threatening arrhythmias over time [1].

PVCs originating from the epicardium have been reported as a risk factor for PVC-induced cardiomyopathy [6,7]. The left ventricular summit (LVS) represents the most superior portion of the left ventricular (LV) epicardium and is an important anatomical region that harbors arrhythmogenic foci responsible for ventricular arrhythmias. PVCs arising from the LVS (LVS-PVCs) often require multiple approaches, including both epicardial and adjacent endocardial sites. Radiofrequency ablation (RFA) for LVS-PVCs typically involves prolonged and high-energy ablation delivered from up to four different anatomical regions. The primary reason for procedural failure in this region is the inability to create durable and effective lesions.

In complex cardiac arrhythmias such as ventricular tachycardia (VT) and atrial fibrillation (AF), increases in cardiac troponin (cTn) levels following RFA have been well documented [8,9,10,11,12,13]. Even in the absence of acute illness, serum cTn levels measured at rest have been shown to be associated with the presence of carotid atherosclerosis, a marker of subclinical cardiovascular dysfunction, independent of confounding factors such as age, sex, body mass index, diabetes, hypertension, and smoking status [14].

However, to the best of our knowledge, there are no data regarding post-procedural changes in cardiac troponin levels in patients undergoing RFA for PVCs. Given the prolonged ablation duration and the need for multiple ablation sites in patients with LVS-PVCs, we hypothesized that successful and effective RFA may be associated with increased myocardial injury and subsequent elevation of cTn levels.

Therefore, the aim of this study was to investigate changes in high-sensitivity cardiac troponin I (hs-cTnI) levels following RFA in patients with LVS-PVCs and to evaluate the potential utility of hs-cTnI as a marker of procedural success.

## 2. Methods

### 2.1. Study Population

In this retrospective cohort study, 1320 patients who underwent electrophysiological study (EPS), three-dimensional (3D) mapping, and radiofrequency ablation (RFA) for drug-refractory symptomatic PVCs in our electrophysiology laboratory between 2017 and 2025 were screened. Patients with symptomatic idiopathic PVCs were eligible if at least one of the following criteria was present: (a) a PVC burden > 20% on 24 h Holter electrocardiography despite medical therapy; (b) documentation of life-threatening arrhythmias on clinical evaluation or 24 h Holter monitoring, including R-on-T phenomenon, sustained or nonsustained ventricular tachycardia (VT), or ventricular fibrillation; or (c) LV ejection fraction (LVEF) < 50%.

A power analysis based on previous studies was performed to determine the required sample size (power 80%, α = 0.05), which indicated that approximately 90 patients would be sufficient for inclusion. After 3D mapping, 132 patients were identified as having LVS-PVCs. Following application of the exclusion criteria, a total of 109 patients were included in the final analysis. Exclusion criteria were age ≤ 18 years; insufficient PVC frequency during the procedure to allow adequate mapping; AF; coronary artery disease; hypertensive heart disease; valvular heart disease; congenital heart disease; severe renal or hepatic disease; electrolyte abnormalities; thyroid dysfunction; presence of short or long QT syndrome; pregnancy or within 3 months postpartum; chronic inflammatory disease; and active malignancy. The study protocol was approved by the Adana City Education and Research Hospital Scientific Research Ethics Committee (approval number: 2025/342/10).

After enrollment, detailed medical history, physical examination findings, and demographic characteristics were recorded. The presence of hypertension, smoking status, heart failure, diabetes mellitus, and hyperlipidemia was documented. Pre-procedural systolic and diastolic blood pressure, heart rate, and laboratory parameters were recorded for all patients. Laboratory assessments included complete blood count, fasting glucose, blood urea nitrogen, creatinine, albumin, total cholesterol, low-density lipoprotein cholesterol, high-density lipoprotein cholesterol, and triglyceride levels. All patients underwent pre-procedural M-mode and two-dimensional transthoracic echocardiographic examinations in the echocardiography laboratory. Measurements were performed independently and blinded by two experienced echocardiography specialists using an EPIQ 7C echocardiography system (Philips Healthcare, Andover, MA, USA). Left ventricular (LV) dimensions were obtained from the parasternal long-axis view by positioning the M-mode cursor immediately beyond the tips of the mitral valve leaflets and perpendicular to the LV long axis. Left ventricular ejection fraction (LVEF) and LV volumes were calculated using the Simpson method from apical two-chamber (A2C) and apical four-chamber (A4C) views [15]. In cases of discrepancy between the two LVEF measurements, the assessment was repeated by an expert echocardiographer, and the final LVEF value was determined by consensus.

### 2.2. 12-Lead Electrocardiographic Evaluation

Pre-ablation 12-lead electrocardiograms (ECGs) demonstrating PVCs were reviewed for all patients. All ECG recordings were obtained in sinus rhythm at a paper speed of 25 mm/s and a standard calibration of 1 mV/10 mm using a MAC 2000 ECG system (GE Medical Systems Information Technologies, Inc., Wauwatosa, WI, USA). All ECGs obtained during sinus rhythm were independently evaluated, blinded to clinical data, by two experienced electrophysiologists (MK and AB), each with more than 10 years of experience in electrophysiology and an annual volume of ≥2000 arrhythmia evaluations.

An established ECG algorithm was used to determine the PVC origin [16]. For LVS-PVC assessment, the presence of right bundle branch block (RBBB) morphology, QRS duration, maximum deflection index (MDI), abrupt R-wave transition in lead V3 (ATV3), and pattern break in lead V2 (PBV2) were analyzed on surface ECGs.

### 2.3. 24 h Holter Electrocardiographic Evaluation

In accordance with guideline recommendations, all patients included in the study underwent 24 h Holter electrocardiography (SEER^TM^ 1000, GE Medical Systems, Milwaukee, WI, USA) prior to RFA to determine PVC burden and to detect the presence of malignant arrhythmias, including R-on-T phenomenon and VT. Holter ECG recordings were independently analyzed by two electrophysiologists (MK and AB) who were blinded to each other’s assessments. PVC burden was defined as the percentage of PVCs relative to the total number of QRS complexes over a 24 h period. Six months after PVC ablation, patients underwent repeat 24 h Holter ECG monitoring for follow-up evaluation.

### 2.4. Measurement of High-Sensitivity Cardiac Troponin I

Fasting venous blood samples for the measurement of high-sensitivity cardiac troponin I (hs-cTnI) were collected between 08:00 and 12:00, 24 h after the RFA procedure. Blood samples were centrifuged at 4000 rpm for 10 min, and serum was separated for analysis. Serum hs-cTnI concentrations were measured using a commercially available enzyme-linked immunosorbent assay (ELISA) kit (Elabscience, Houston, TX, USA). Absorbance readings were obtained with an automated ELISA reader (Thermo Scientific, Vantaa, Finland) and analyzed using dedicated software (ScanIt for Multiscan FC, version 2.5.1). The analytical sensitivity of the hs-cTnI assay was 0.38 ng/L, with a measurable range of 0.63–40 ng/L. The intra-assay coefficient of variation was <10%, and results were expressed in ng/L.

### 2.5. Electrophysiological Study and Radiofrequency Ablation Protocol

All patients underwent EPS after discontinuation of antiarrhythmic drugs used for palpitations or PVCs for at least five half-lives. EPS procedures were performed using the WorkMate Claris^TM^ system (St. Jude Medical, St. Paul, MN, USA). Both right and left inguinal regions were prepared for vascular access. Via the left femoral vein, a quadripolar diagnostic catheter was positioned in the high right atrium and a decapolar catheter in the coronary sinus. Through the right femoral vein, a quadripolar diagnostic catheter was placed in the right ventricular (RV) apex. In addition, an irrigated ablation catheter (Thermocool SmartTouch SF or Thermocool SF; Biosense Webster, Diamond Bar, CA, USA, or TactiCath; Abbott, Abbott Park, IL, USA) was introduced via femoral arterial or venous access using a long sheath.

Mapping of the RV outflow tract (RVOT) and RV was performed using a standard femoral approach. The left ventricle (LV) and LV outflow tract (LVOT) were mapped via a retrograde transaortic approach. During ablation procedures, intravenous heparin was administered at a dose of 50–100 IU/kg according to body weight, and the procedure was continued with an activated clotting time maintained between 300 and 500 s. If PVCs were infrequent or VT could not be induced in patients with a prior history of VT, intravenous isoproterenol infusion (1–5 μg/min) was administered. RFA was continued when sufficient PVCs or VT were present.

Three-dimensional electroanatomical mapping systems (CARTO 3, Biosense Webster, Diamond Bar, CA, USA, and EnSite X, Abbott, Abbott Park, IL, USA) were used to map PVCs and VT. After adequate mapping and point acquisition, areas showing the earliest local electrical activation on bipolar mapping and a QS pattern on unipolar mapping were identified and tagged.

**Figure 1:**
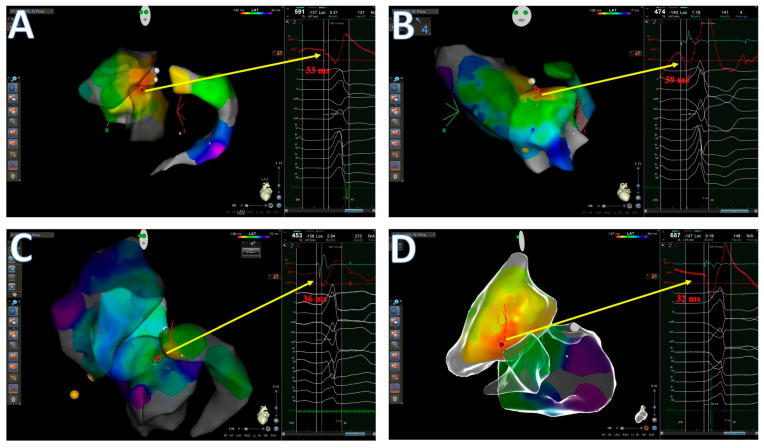
Activation mapping of symptomatic premature ventricular complexes originating from the left ventricular summit (LVS) in four representative patients. (**A**) The earliest activation site was localized at the left coronary cusp (LCC) on the aortic sinus of Valsalva side (earliest activation time [EAT]: 33 ms pre−QRS). (**B**) The earliest activation site was localized at the subvalvular region of the LCC (EAT: 39 ms pre−QRS). (**C**) The earliest activation site was localized at the distal great cardiac vein (GCV) (EAT: 36 ms pre−QRS). (**D**) The earliest activation site was localized at the septal region of the right ventricular outflow tract (RVOT) (EAT: 32 ms pre−QRS).

**Figure 2:**
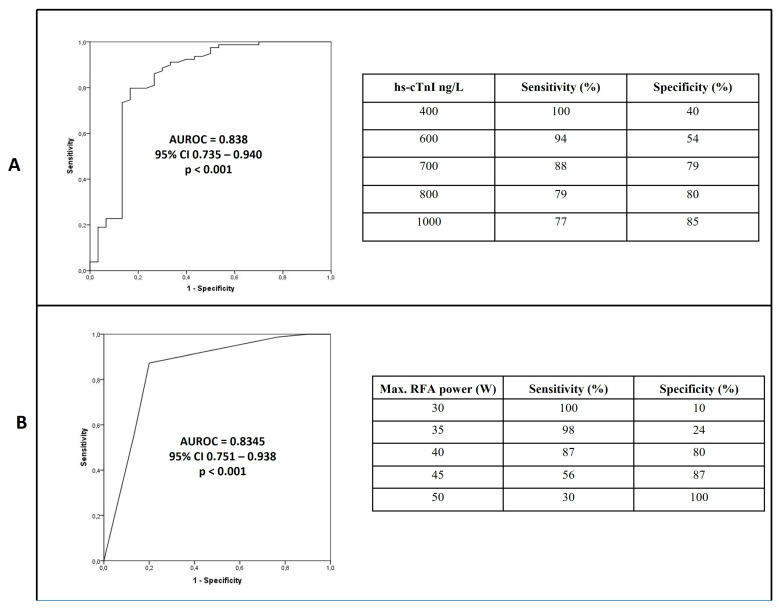
Receiver operating characteristic (ROC) curves and cutoff values of high-sensitivity cardiac troponin I (**A**) and maximum RFA power (**B**) for predicting RFA success.

In patients in whom the earliest activation site for PVCs was localized to the LVS, repeat 3D mapping was performed from four distinct anatomical regions: (1) the left coronary cusp (LCC) at the aortic sinus of Valsalva (Figure 1A); (2) the subvalvular region of the LCC (Figure 1B); (3) the great cardiac vein/anterior interventricular vein (GCV/AIV) accessed via the coronary sinus (Figure 1C); and (4) the septal region of the RVOT (Figure 1D). Subsequently, multisite RFA was initiated, starting from the region with the absolute earliest activation time, and ablation was delivered to the earliest activation sites in all four anatomical regions.

Before delivering RFA within the GCV/AIV or LCC–aortic sinus of valsalva regions, coronary angiography was performed to assess the proximity to the coronary arteries, and a minimum distance of >5 mm was ensured. Ablation was performed using irrigated RF catheters (Thermocool Smarttouch SF [Biosense Webster, Diamond Bar, CA, USA] and TactiCath [Abbott, Abbott Park, IL, USA]) with power settings ranging from 30 to 50 W, depending on tissue characteristics and adjacent cardiac structures, and was continued until PVCs were eliminated for more than 3 min and local impedance decreased by <10 ohms. During ablation within the GCV and AIV, irrigated RF catheters were used in all patients. The RF power was set at 30 W, with an irrigation flow rate of 15 mL/min. Finally, the presence of residual PVCs or VT was reassessed using isoproterenol infusion and programmed stimulation maneuvers.

### 2.6. Definition of Radiofrequency Ablation Success

Acute procedural success for PVC RFA was defined as the complete elimination of previously observed frequent PVCs during the procedure, with no inducibility of the targeted PVCs. All patients were followed after radiofrequency ablation, with the first follow-up visit scheduled at 1 month and subsequent visits at 3-month intervals. At each follow-up, patients underwent clinical evaluation, including medical history, physical examination, assessment of symptom recurrence, and electrocardiographic analysis. In addition to routine clinical assessment, all patients underwent 24 h Holter electrocardiography and transthoracic echocardiography at 1, 3, and 6 months after the procedure. Recurrence was defined as the presence of more than 1000 PVCs or a PVC burden > 1% on 24 h Holter monitoring, or the documentation of sustained or nonsustained ventricular tachycardia [17]. Patients who did not meet any of these recurrence criteria were classified as having successful RFA.

### 2.7. Statistical Analysis

Statistical analyses were performed using SPSS 23.0 version (SPSS for Windows, Chicago, IL, USA). Continuous variables are presented as mean ± standard deviation or median and interquartile range, and categorical variables are expressed as numbers and percentages. The normality of continuous variables was assessed using the Kolmogorov–Smirnov test. Inter–intraobserver variability were evaluated using the kappa coefficient. Comparisons between two groups were performed using the Student’s *t*-test for normally distributed continuous variables and the Mann–Whitney U test for non-normally distributed variables. Categorical variables were compared using the chi-square test. Logistic regression analysis was conducted to identify independent predictors of RFA success. Variables with a *p*-value < 0.25 in the univariable analysis were subsequently entered into the multivariable model using a stepwise forward selection procedure. In logistic regression analyses, non-parametric variables were categorized and included in the model as categorical variables. Receiver operating characteristic (ROC) curve analysis was subsequently performed for variables independently associated with RFA success, and sensitivity and specificity values for predicting recurrence were calculated. A *p* value < 0.05 was considered statistically significant for all analyses.

## 3. Results

A total of 109 patients who underwent LVS-PVCs were included in the study (44 women, 65 men; mean age 53.9 ± 9.8 years). Acute procedural success was achieved in 90 patients (82.6%). At 6-month follow-up, long-term procedural success based on Holter ECG evaluation was observed in 79 patients (72.5%). Six patients (5.5%) did not demonstrate acute procedural success but showed delayed procedural success at 24 h after ablation; none of these patients experienced long-term recurrence during follow-up.

Cohen’s kappa coefficients assessing interobserver variability exceeded 0.90 for all ECG, LVEF and Holter ECG parameters (*p* < 0.001 for all comparisons). Serum hs-cTnI levels increased in all patients following RFA for LVS-PVCs. Post-procedural hs-cTnI values ranged from 132 to 4312 ng/L, with a median of 1276 ng/L (interquartile range [IQR]: 772–2249) and a mean value of 1555 ± 1058 ng/L.

Patients with LVS-PVCs were subsequently divided into two groups according to long-term RFA outcome (successful vs. unsuccessful), and all clinical, electrocardiographic, and procedural parameters were compared between the groups.

### 3.1. Baseline Demographic, Clinical, Laboratory, and Medical Treatment Characteristics of the Patient Groups

The demographic, clinical, and laboratory characteristics of patients with successful and unsuccessful RFA are summarized in Table 1. Serum hs-cTnI levels were significantly higher in patients with successful RFA compared with those with unsuccessful procedures. Other demographic, clinical, and laboratory parameters were similar between the two groups (Table 1).

In the overall study population, 27 patients (25%) had a LVEF < 50%. The proportion of patients with LVEF < 50% did not differ significantly between the successful and unsuccessful RFA groups.

### 3.2. Pre-Ablation Electrocardiographic, Electrophysiological, and Ablation Characteristics of Patients with Successful and Unsuccessful RFA

Pre-ablation electrocardiographic, EPS, and ablation characteristics of patients with successful and unsuccessful RFA are presented in Table 2. On pre-ablation ECG analysis, the presence of RBBB morphology and longer QRS duration were significantly more frequent in patients with successful RFA, whereas the MDI and the presence of a PBV2 were significantly lower (Table 2).

Patients with successful RFA more frequently required ablation at the LCC–ASV, the subvalvular region of the LCC, the GCV/AIV, and multiple ablation sites. In addition, maximum RFA power and the rate of acute procedural success were significantly higher in the successful RFA group (Table 2). Other pre-ablation ECG, EPS, and ablation parameters were similar between the two groups (Table 2).

### 3.3. Independent Predictors of Successful Radiofrequency Ablation

To identify variables independently associated with successful RFA among patients with LVS-PVCs, a multivariable logistic regression analysis was performed, including all parameters associated with RFA success. This analysis demonstrated that maximum RFA power, hs-cTnI level, and ablation within the GCV/AIV were independently associated with successful RFA (Table 3).

Specifically, each 1 W increase in maximum RFA power, each 100 ng/L increase in hs-cTnI level, and the presence of GCV/AIV ablation were associated with a 44.4%, 13.3%, and 3.28-fold increase in the likelihood of RFA success, respectively (Table 3).

### 3.4. ROC Curve Analysis of Independent Predictors of RFA Success

Receiver operating characteristic (ROC) curve analysis was performed to evaluate the ability of maximum RFA power and hs-cTnI levels to discriminate successful RFA. Both parameters were found to be significant predictors of RFA success, with areas under the ROC curve (AUCs) of 0.845 and 0.838, respectively (Figure 2A,B). Different cutoff values of maximum RFA power and hs-cTnI levels yielded varying sensitivities and specificities for predicting successful RFA. These cutoff points and their corresponding sensitivity and specificity values are presented in Figure 2A,B.

## 4. Discussion

To the best of our knowledge, this study is the first to demonstrate that hs-cTnI levels increase after RFA in patients with LVS-PVCs and that this increase is associated with procedural success. Our findings yield several important conclusions: (1) hs-cTnI levels increase in all patients undergoing RFA for LVS-PVCs; (2) hs-cTnI levels are significantly higher in patients with successful RFA compared with those with unsuccessful procedures; (3) elevated hs-cTnI levels are independently associated with RFA success; and (4) an hs-cTnI level of 700 ng/L measured 24 h after RFA may serve as a cutoff value for predicting procedural success in this patient population.

The biological effect of RFA is mediated through thermal injury of myocardial tissue at the catheter–tissue interface, resulting in localized myocardial damage that enables effective and durable lesion formation [18]. Reichlin et al. [8] previously reported significant increases in hs-cTnT and hs-cTnI levels measured at 30, 60, 90, and 120 min, as well as at 4 and 24 h, following catheter ablation for complex arrhythmias such as VT and AF. In that study, troponin elevation occurred more rapidly in patients with AF; however, 24 h troponin levels were comparable between VT and AF [8]. Reichlin et al. also reported a median hs-cTnI level of 2165 ng/L (IQR: 1848–4756) in patients with VT, with troponin elevation correlating with the extent of delivered ablation energy. In our cohort, the median hs-cTnI level after LVS-PVC ablation was 1276 ng/L (IQR: 772–2249), which is lower than that reported in VT ablation. This difference is likely attributable to the more complex nature of VT, which typically requires more extensive and aggressive ablation over a broader myocardial area.

Several studies have investigated post-ablation changes in cTn levels, particularly in patients undergoing AF ablation [8,9,10,11,12,13], whereas data in VT populations remain limited [8]. In patients with AF, elevated post-procedural hs-cTnT levels have been associated with procedural success and left atrial remodeling, likely reflecting greater myocardial injury to viable atrial tissue [9]. Other studies have shown that cryoballoon ablation results in lower troponin release compared with RFA [12]. Furthermore, cTn levels have been reported to increase up to 20-fold after AF ablation, suggesting that troponin elevation in this context does not represent myocardial ischemia [13]. To our knowledge, no prior studies have evaluated cTn changes after RFA in patients with PVCs. Therefore, our study is clinically relevant, as it demonstrates a significant increase in hs-cTnI following RFA in a specific PVC population and confirms that higher hs-cTnI levels are associated with successful LVS-PVC ablation, similar to findings reported in AF ablation.

Previous studies have reported acute procedural success rates of 80–94% and complication rates of approximately 5.6% for RFA in patients with PVCs [19,20,21]. However, in LVS-PVCs, acute success rates are generally lower and highly variable (22–100%) due to anatomical challenges, increased myocardial thickness, and the predominantly epicardial origin of these arrhythmias [22,23,24,25,26]. Kuo et al. [22] demonstrated a procedural success rate of 92.8% using comprehensive 3D mapping and RFA from the LCC-ASV, LCC subvalvular region, GCV/AIV, epicardial sites, and areas of earliest activation, with multisite ablation when necessary. In our study, mapping and ablation were performed from three similar regions—LCC–ASV, the subvalvular LCC region, and the GCV/AIV—with the addition of RVOT septal mapping and ablation instead of a direct epicardial approach. A substantial proportion of patients underwent multisite ablation. Acute and long-term procedural success rates were 82.6% and 72.5%, respectively. Among several factors associated with RFA success, elevated hs-cTnI levels at 24 h, higher maximum RFA power, and ablation within the GCV/AIV were independently associated with successful outcomes. These findings suggest that the use of higher energy delivery during LCC–ASV ablation and adjunctive ablation at the GCV/AIV earliest activation site may improve procedural success.

Idiopathic PVCs are typically related to triggered activity and delayed afterdepolarizations, particularly in arrhythmias originating from the RVOT and LVOT [1]. In patients without structural heart disease, idiopathic PVCs are generally benign and do not usually result in early structural or functional cardiac deterioration [1]. Nevertheless, a subset of patients may develop reduced LVEF, syncope, hypotension, VT, implantable cardioverter-defibrillator requirement, ventricular fibrillation, or sudden cardiac death [1,27]. Haïssaguerre et al. [27] reported that apparently benign outflow tract PVCs may cause malignant arrhythmias even in the absence of underlying structural heart disease, channelopathies, or electrolyte imbalance. The reported prevalence of PVC-induced cardiomyopathy is 7–10% in the general PVC population [28,29,30,31], whereas a substantially higher prevalence of 28.8% has been described in patients with LVS-PVCs [20]. Consistent with prior reports, PVC-induced cardiomyopathy was observed in 25% of patients in our cohort.

### Limitations

This study has several important limitations. First, it was a single-center, retrospective analysis with a relatively small sample size. Second, only patients with LVS-PVCs were included, and those with PVCs secondary to structural heart disease were excluded, which may limit the generalizability of the findings. Third, because multisite RFA was performed in most cases, we were unable to determine which specific anatomical ablation site was primarily responsible for the observed troponin elevation. In addition, the study population consisted exclusively of patients undergoing RFA, and no comparison group receiving medical therapy was included. Finally, due to the relatively short follow-up period, we did not evaluate longer-term outcomes such as improvement in LVEF or prognosis, which have been reported in previous studies [22]. Novel RF ablation catheters are designed with flexible porous-tip electrodes to facilitate saline irrigation during the ablation procedure. The flexible tip may reduce procedural risks by directing and maintaining a uniform irrigation flow and may potentially support deeper lesion formation through an irrigated circuit. Compared with fixed-tip catheters, the flexible-tip design can achieve better tissue contact without causing additional trauma to the cardiac tissue and may increase the effective surface area of catheter–tissue contact. These advantages may contribute to improved RF ablation procedural success [32,33]. In our study, this novel catheter technology was not utilized. The use of these advanced catheters may have potentially resulted in improved procedural outcomes.

## 5. Conclusions

In patients with LVS-PVCs, hs-cTnI levels increase after RFA. Higher hs-cTnI levels are independently associated with procedural success. Measurement of hs-cTnI at 24 h after RFA may serve as an objective and noninvasive marker for predicting procedural success in this patient population. However, future large-scale and long-term prospective studies are warranted to further investigate the biomarker role and clinical utility of elevated troponin levels measured after ablation of LV summit-originating PVCs in predicting intramural lesion depth, durable arrhythmia suppression, and late recurrence rates.

## Figures and Tables

**Table 1:** Pre-ablation demographic, clinical, and laboratory characteristics of patients with successful and unsuccessful RFA.

Variables	Patients with Successful RFA *n* = 79	Patients with Unsuccessful RFA *n* = 30	*p*
Age (year)	54.2 ± 9.4	53.4 ± 10.9	0.735
Gender (Male), n	47 (60%)	18 (60%)	1.000
Hypertension, n (%)	28 (35%)	14 (47%)	0.378
Diabetes, n (%)	13 (17%)	8 (27%)	0.278
Smoking, n (%)	11 (14%)	5 (17%)	0.765
BMI (kg/m^2^)	28.4 ± 3.7	27.8 ± 4.2	0.492
SBP (mmHg)	125 ± 22	126 ± 24	0.799
DBP (mmHg)	79 ± 20	78 ± 21	0.845
Heart rate (beat/min)	72.9 ± 8.4	75.3 ± 8.4	0.192
Beta blocker, n (%)	50 (63%)	18 (60%)	0.735
Calcium channel blocker, n (%)	15 (19%)	6 (20%)	0.815
Class I AAD, n (%)	25 (32%)	12 (40%)	0.498
Amiodarone, n (%)	8 (10%)	4 (13%)	0.377
WBC count (10^3^/µL)	8.93 ± 2.1	8.58 ± 2.4	0.465
Platelet count (10^3^/µL)	239 ± 58	226 ± 47	0.299
Hemoglobin (g/dL)	14.3 ± 2.1	13.9 ± 3.3	0.500
Glucose (mg/dL)	101 ± 22	105 ± 25	0.414
BUN (mg/dL)	33.2 ± 11.1	33.3 ± 9.4	0.982
Creatinine (mg/dL)	0.88 ± 0.25	0.89 ± 0.22	0.694
Total cholesterol (mg/dL)	195 ± 41	195 ± 37	0.967
LDL (mg/dL)	133 ± 31	127 ± 29	0.320
HDL (mg/dL)	49 ± 10	48 ± 7	0.564
Triglycerides (mg/dL)	144 ± 71	150 ± 86	0.724
AST (u/L)	25.6 ± 14.5	23.4 ± 8.6	0.431
ALT (u/L)	26.3 ± 18.5	29.5 ± 12.4	0.444
Hs-Troponin I level (ng/L)	1567 (1096–2345)	523 (294–977)	**<0.001**
LVEF (%)	52.7 ± 5.3	52.5 ± 6.9	0.805
LVEF < 50%, n (%)	17 (22%)	10 (33%)	0.221

The values were shown as mean ± standard deviation, median and interquartile range and n (%), Statistically significant p values were shown in bold. AAD: Antiarrhythmic drug, ALT: Alanine aminotransferase, AST: Aspartate aminotransferase, BMI: Body mass index, BUN: Blood urea nitrogen, DBP: Diastolic blood pressure, HDL: High-density lipoprotein, LDL: Low-density lipoprotein, LVEF: Left ventricular ejection fraction, RFA: Radiofrequency ablation, SBP: Systolic blood pressure, WBC: White blood cell.

**Table 2:** Pre-ablation electrocardiographic, electrophysiological, and ablation characteristics of patients with successful and unsuccessful RFA.

Variables	Patients with Successful RFA *n* = 79	Patients with Unsuccessful RFA *n* = 30	*p*
PVC burden (preablation), %	19.9 ± 3.4	20.8 ± 3.4	0.222
Sustained VT, n (%)	11 (14%)	7 (23%)	0.257
RBBB morphology, n (%)	37 (47%)	4 (13%)	**0.002**
QRS duration, ms	171 ± 7.4	166 ± 7.6	**0.005**
Maximum deflection index	0.50 ± 0.03	0.53 ± 0.004	**<0.001**
ATV3, n (%)	14 (18%)	2 (7%)	0.226
PBV2, n (%)	6 (7%)	6 (20%)	**0.042**
Earliest activation before VA, ms	31.1 ± 4.1	29.9 ± 3.2	0.215
LCC-ASV mapping, n (%)	79 (100%)	28 (93%)	0.074
LCC subvalvular mapping, n (%)	77 (98%)	27 (90%)	0.127
GCV/AIV mapping, n (%)	78 (99%)	27 (90%)	0.063
RVOT mapping, n (%)	47 (66%)	24 (34%)	0.071
LCC-ASV RFA, n (%)	77 (98%)	26 (87%)	**0.027**
LCC subvalvular RFA, n (%)	65 (82%)	5 (17%)	**<0.001**
GCV/AIV RFA, n (%)	29 (37%)	1 (3%)	**<0.001**
RVOT RFA, n (%)	27 (34%)	16 (53%)	0.069
Multiple site RFA requirement 2–3–4, n	35–30–8	14–2–0	**<0.001**
Maximum RFA power (W)	47.1 ± 3.7	40.1 ± 5.4	**<0.001**
Total RFA duration, seconds	620 ± 226	339 ± 238	**<0.001**
Acute procedural success, n (%)	73 (92%)	17 (57%)	**<0.001**
Late procedural success, n (%)	6 (8%)	0 (0%)	0.123

The values were shown as mean ± standard deviation and n (%), Statistically significant p values were shown in bold. AIV: Anterior interventricular vein, ASV: Aortic sinus Valsalva, ATV3: Abrupt R-wave transition in lead V3, GCV: Great cardiac vein, LCC: Left coronary cusp, PBV2: Pattern break in V2, RBBB: Right bundle branch block, RFA: Radiofrequency ablation, RVOT: Right ventricular outflow tract.

**Table 3:** Multivariable logistic regression analysis for predictors of RFA success in patients with LVS-PVCs.

	Odds Ratio	95% CI	*p*
Maximum RFA power (each 1 W)	1.444	1.176–1.773	**<0.001**
hs-cTnI level (each 100 ng/L)	1.133	1.043–1.231	**0.003**
GCV/AIV RFA (presence)	3.281	1.325–21.219	**0.007**

Statistically significant p values were shown in bold. AIV: Anterior interventricular vein, GCV: Great cardiac vein, hs-cTnI: High-sensitive cardiac troponin I, RFA: Radiofrequency ablation.

## Data Availability

The data that support the findings of this study are available from the corresponding authors upon reasonable request.
